# Evaluating the Role of *MIA3* Variant rs17465637 in Coronary Artery Disease: A Comprehensive Case–Control Analysis

**DOI:** 10.3390/diagnostics16162489

**Published:** 2026-08-07

**Authors:** Neda M. Bogari, Samar N. Ekram, Amr A. Amin, Mashhour S. Alotaibi, Naif A. Almalki, Samar A. Amer, Rami Obaid, Reem M. Allam

**Affiliations:** 1 Department of Medical Genetics, Faculty of Medicine, Umm Al-Qura University, Makkah 24382, Saudi Arabia; nmbogari@uqu.edu.sa (N.M.B.); snekram@uqu.edu.sa (S.N.E.); 2 Department of Medical Biochemistry, Faculty of Medicine, Umm Al-Qura University, Makkah 24382, Saudi Arabia; aaamin@uqu.edu.sa; 3 Faculty of Medicine, Ain-Shams University, Cairo 11566, Egypt; 4 Al-Hada Armed Forces Hospital, Taif 26792, Saudi Arabia; mashhour_saud@hotmail.com; 5 King Faisal Medical Complex, Taif 26521, Saudi Arabia; nalmalki1@moh.gov.sa; 6 Department of Public Health and Community Medicine, Faculty of Medicine, Zagzig University, Zagazig 44519, Egypt; dr_samar11@yahoo.com; 7 Department of Medical Genetics, Faculty of Medicine, Umm Al-Qura University, Al-Qunfudah 21912, Saudi Arabia; raaobaid@uqu.edu.sa; 8 Department of Clinical Pathology, Faculty of Medicine, Zagazig University, Zagazig 44519, Egypt

**Keywords:** CAD, *MIA3*, *rs17465637*, Saudi population, SNPs

## Abstract

**Objectives:** Coronary artery disease (CAD) remains a leading cause of morbidity and mortality worldwide, yet population-specific evidence regarding the contribution of *MIA3* genetic variation in Middle Eastern populations remains limited. This study investigated the association of the *MIA3* rs17465637 polymorphism with CAD susceptibility and its relationship with lipid-related phenotypes in a Saudi population. **Methods**: A case–control study was conducted between June 2020 and August 2022, including 200 patients with angiographically confirmed CAD and 200 age- and sex-matched healthy Saudi controls. Genotyping of rs17465637 was performed using a TaqMan real-time polymerase chain reaction assay. Genotype distributions were evaluated using chi-square analysis under multiple inheritance models. Multivariable logistic regression was subsequently performed to estimate adjusted odds ratios after controlling age, BMI, smoking, physical inactivity, systolic BP, diastolic BP, blood glucose, triglycerides, total cholesterol, LDL-C, and HDL-C. Associations between rs17465637 genotypes and serum lipid parameters were also examined. **Results**: Genotype frequencies of rs17465637 differed modestly between cases and controls; however, unadjusted comparisons under codominant, dominant, recessive, and allelic inheritance models did not reach statistical significance, and none remained significant after Bonferroni correction. In contrast, multivariable logistic regression demonstrated an independent association between the rs17465637 C allele and CAD after adjustment for conventional cardiovascular risk factors. In genotype–phenotype analyses, carriers of the C allele exhibited higher association with low-density lipoprotein cholesterol concentrations and less favorable lipid profiles than AA homozygotes, supporting a relationship between the variant and lipid metabolism. These findings are consistent with a potential contribution of rs17465637 to CAD susceptibility through lipid-related pathways. **Conclusions**: Although unadjusted genotype comparisons were not statistically significant after correction for multiple testing, multivariable analysis provides evidence supporting an independent association between the *MIA3* rs17465637 variant and CAD susceptibility in this Saudi cohort. The observed associations with adverse lipid profiles further provide evidence linking this specific locus to the molecular mechanisms underlying cardiovascular disease. Replication in larger, multi-center studies incorporating genome-wide ancestry-informative markers and functional investigations is warranted to further minimize the possibility of residual population stratification, confirming these findings and clarifying the biological mechanisms underlying this association.

## 1. Introduction

The most common heart condition is coronary artery disease (CAD), which is brought on by atheroma plaque formation and coronary artery stenosis. 45% of deaths worldwide are attributable to cardiovascular disease, with half of those deaths occurring in Asia [1,2,3]. A study conducted in late 2020 by the World Health Organization (WHO) revealed that the number of deaths in the Western Pacific region (which includes the Philippines), attributed to ischemic heart diseases, rose from 1 million in 2000 to 2.3 million in 2019 [4].

CAD is an increasingly pressing public health issue in the Gulf Council countries, including Saudi Arabia, where it is estimated that CAD accounts for over 45% of all deaths [5,6,7]. In the North Africa and Middle East regions, mortality rates for heart disease are 115 and 294 per 100,000, respectively [6].

It has multiple causes, and while certain families are more affected than others, the exact route of inheritance is unknown. CAD may result from the interaction of environmental factors and hereditary characteristics. In the past, a number of risk variables have only been able to explain a small percentage of the reported cases [8]. The principal mechanism of CAD is the reduction in blood flow in the epicardial coronary arteries due to blockage by atherosclerotic plaques (atherosclerotic CAD) [9,10,11].

In asymptomatic individuals, coronary artery calcification has been found [12] to be a strong predictor of mortality. Despite progress in treatment, coronary artery disease (CAD) continues to be associated with significant morbidity and mortality on a global scale [13,14] with approximately 30% of deaths worldwide attributed to CAD [6,14,15,16].

Since the completion of multiple massive genome-wide association studies (GWAS) that examined DNA variation throughout the whole human genome, substantial progress has been made in understanding the genetic basis of CAD [17].

Additionally, chromosomal region 1q41 represents a significant locus associated with coronary artery disease (CAD) risk. The strongest association signal within this locus maps to the single-nucleotide polymorphism (SNP) rs17465637, located within the melanoma inhibitory activity 3 (*MIA3*) gene. Also referenced in literature as *TANGO1*, *ARNT*, *D320*, *ODCD2*, *TANGO*, and *UNQ6077*, *MIA3* exhibits a ubiquitous tissue expression pattern [18,19,20,21]. In addition, *MIA3* may be able to increase CAD risk through inflammatory processes. Numerous studies provide evidence for the crucial role those inflammatory mechanisms of action play in the formation of atheroma [18].

The phenotypic diversity observed in CAD patients is most likely caused by the interaction between the environmental factors and the complicated pathophysiological pathways in the human body. These pathways involve the interactions between distinct genetic variables encoding different molecules. It is highly desirable to identify the risk alleles that provide a higher susceptibility to CAD [9].

The current clinical risk assessment of CAD mostly uses conventional risk variables, giving genetic factors little significance. This approach may lead to an underestimation of risk and a failure to identify possibilities for disease prevention in at-risk patients [19]. SNPs enhance risk prediction over a 15-year follow-up when included in risk assessment, indicating the necessity of genetic analysis to determine an individual’s vulnerability to developing CAD [20].

The purpose of our research is to ascertain whether SNP rs17465637 is associated with the risk of CAD and post-myocardial infarction in the Saudi Arabian population and correlate this genetic variant with various disease risk factors. To our knowledge, this research represents the first investigation of this SNP in Saudi Arabia.

## 2. Materials and Methods

### 2.1. Study Population

This multicenter case–control study recruited Saudi participants from King Abdullah Medical City (KAMC) and Al-Noor Specialist Hospital, Makkah, Saudi Arabia, between June 2020 and August 2022. The study was conducted in accordance with the principles of the Declaration of Helsinki and was approved by the Ethics Committee of the Saudi Ministry of Health, Makkah Region (Approval No. H-02-K-076-0324-1099; approval date: 28 April 2020). Written informed consent was obtained from all participants prior to enrollment.

The required sample size was estimated using OpenEpi for an unmatched case–control design. The calculation assumed a two-sided 95% confidence level, 80% statistical power, a 1:1 case-to-control ratio, an expected exposure frequency of 50% among controls, and a minimum detectable odds ratio of 1.8. The estimated minimum sample size was 374 participants, comprising approximately 187 cases and 187 controls. After allowing for approximately 5% incomplete or unusable data, the target sample size was increased to 394 participants. The final study included 400 participants, consisting of 200 patients with coronary disease and 200 controls, and therefore met the estimated sample-size requirement.

The case group consisted of 200 patients with coronary artery disease (CAD), including 100 patients with angiographically confirmed CAD and 100 patients with post-myocardial infarction (PMI). CAD was diagnosed according to established clinical and cardiological criteria, including typical ischemic symptoms together with at least one of the following: documented coronary artery disease, elevated cardiac biomarkers, electrocardiographic evidence of myocardial ischemia (ST-segment elevation or depression, T-wave inversion, or new left bundle branch block), or angiographic confirmation of significant coronary artery stenosis, as determined by a consultant cardiologist. Patients with congenital heart disease, cardiomyopathy, clinically significant valvular heart disease, advanced hepatic or renal disease, or other severe systemic comorbidities were excluded.

The PMI subgroup comprised patients presenting with acute ST-segment elevation myocardial infarction (STEMI), diagnosed by characteristic ischemic symptoms accompanied by ST-segment elevation in at least two contiguous electrocardiographic leads. To establish a precise chronological window for the index event and ensure a direct, predictable correlation with infarct size—thereby mitigating the prolonged elevation kinetics associated with cardiac troponins—enzymatic confirmation required a peak plasma creatine kinase (CK) elevation to at least twofold the upper limit of normal (≥400 U/L). Eligibility was further restricted to patients surviving a minimum of 24 h post-myocardial infarction to enable baseline enzymatic tracking. Patients presenting cardiogenic shock or aged ≥80 years were excluded, as these conditions substantially influence clinical and biochemical phenotypes independent of genetic susceptibility. Crucially, given that the primary objective of this study was to evaluate baseline genetic susceptibility to CAD rather than differences in acute or chronic disease presentation or severity, stable CAD (n=100) and PMI (n=100) subgroups were analyzed as a unified case–cohort (n=200) to maximize statistical power for genetic association testing.

The control group comprised 200 apparently healthy Saudi individuals recruited from regional blood donation services. Controls were free of clinically documented cardiovascular disease according to predefined screening criteria, as confirmed through clinical evaluation, resting electrocardiography, and review of medical records. With no malignancy or pregnancy. Anthropometric measurements were obtained using standardized procedures, and body mass index (BMI) was calculated as weight (kg) divided by height squared (m^2^). Hypertension was defined as a previous physician diagnosis with ongoing antihypertensive treatment or a resting systolic blood pressure ≥140 mmHg and/or diastolic blood pressure ≥90 mmHg. Demographic characteristics, cardiovascular risk factors, medication history, and clinical information were collected using standardized interviews and structured questionnaires.

To reduce the potential influence of population stratification, all participants were self-reported Saudi individuals of Arab ancestry recruited from the same geographical region using identical eligibility criteria and standardized recruitment procedures for both cases and controls. Individuals with chronic hepatic or renal disease, autoimmune disorders, active malignancy, pregnancy, or medications known to substantially affect lipid metabolism were excluded from both groups.

### 2.2. Echocardiography

Patients with CAD and PMI underwent transthoracic echocardiography utilizing an ATL HDI 5000 (Philips Healthcare; Andover, MA, USA) at KAMC Hospital and a GE Vivid 3 ultrasound system (GE Medical Systems; Waukesha, WI, USA) at Al-Noor Hospital. In accordance with the American Society of Echocardiography [17], apical 4- and 2-chamber views were part of the imaging methodology. A cardiology consultant evaluated the results. For M-mode measurements of left ventricular (LV) dimensions, parasternal and short- and long-axis images (averaged over 4 cycles) were acquired. Planimetry was used to calculate the biplane diastolic and systolic volumes as well as the ejection fraction using the Simpson method.

### 2.3. Blood Sample Collection

A 5-milliliter sample of venous blood was extracted from patients and control groups under complete aseptic conditions and following International Federation of Clinical Chemistry and Laboratory Medicine (IFCC) guidelines for possible COVID-19 infection [21]. An enzymatic colorimetric approach was used for biochemical analysis of the amounts of lipids, total cholesterol, and blood glucose. For DNA extraction, a 5-milliliter sample of peripheral blood was obtained and placed in tubes containing EDTA.

### 2.4. Assay Design and Reagent Preparation

The MIA3 rs17465637 single-nucleotide polymorphism (SNP) is an intronic genetic variant located within the *MIA3* (Melanoma Inhibitory Activity Family Member 3; also known as *TANGO1*) gene on chromosome 1q41. The variant lies in a non-coding regulatory region and has been identified by genome-wide association studies (GWAS) as a susceptibility locus for coronary artery disease (CAD) and myocardial infarction (MI). The rs17465637 polymorphism was genotyped using a predesigned TaqMan^®^ SNP Genotyping Assay (Applied Biosystems, Foster City, CA, USA) based on allele-specific fluorogenic probe chemistry. The assay incorporates two sequence-specific TaqMan^®^ minor groove binder (MGB) probes labeled with distinct fluorescent dyes, VIC^®^ for the reference allele and FAM™ for the alternate allele, enabling simultaneous discrimination of both alleles within a single PCR reaction. The incorporation of MGB chemistry enhances probe-binding specificity and improves discrimination between alleles differing by a single nucleotide, thereby providing reliable genotype assignment.

Reaction mixtures were prepared in a dedicated pre-amplification laboratory using aerosol-resistant filtered pipette tips to minimize the risk of contamination. Each 10-μL reaction contained 2× TaqMan^®^ Genotyping Master Mix, the rs17465637-specific TaqMan^®^ SNP Genotyping Assay at the manufacturer’s recommended final concentration (1×), and 20 ng of genomic DNA template. Reaction plates were sealed with optical adhesive film, briefly centrifuged to ensure complete mixing and remove air bubbles, and subsequently loaded into the real-time PCR instrument for amplification and allelic discrimination analysis.

### 2.5. Thermal Cycling Conditions

PCR amplification and allelic discrimination were performed on a real-time PCR platform (Applied Biosystems, Thermo Fisher Scientific, Waltham, MA, USA) using the manufacturer’s recommended TaqMan^®^ SNP Genotyping protocol. Thermal cycling consisted of an initial enzyme activation step at 95 °C for 10 min, followed by 40 cycles of denaturation at 92 °C for 15 s and combined annealing and extension at 60 °C for 60 s. Fluorescence signals were acquired during each annealing–extension cycle. Following amplification, genotype calls were assigned using the allelic discrimination module of the instrument software, which generated fluorescence cluster plots for automated genotype classification.

### 2.6. Quality Control and Genotype Calling

Quality-control procedures were implemented throughout the genotyping workflow in accordance with the STREGA recommendations for reporting genetic association studies. Each 96-well reaction plate included at least two no-template controls containing nuclease-free water to monitor reagent contamination and non-specific amplification. Samples with previously confirmed genotypes were incorporated as internal positive controls to assess assay reproducibility across experimental runs. Reaction plates demonstrating evidence of contamination, poor amplification efficiency, or inadequate fluorescence clustering were repeated before genotype assignment.

Genotype calls were generated automatically using the allelic discrimination module of the StepOne™ Real-Time PCR software v 2.1 based on endpoint fluorescence cluster analysis and were subsequently verified by visual inspection of the fluorescence cluster plots. Samples producing ambiguous or low-intensity fluorescence signals were reanalyzed, and only samples with unequivocal genotype assignments were retained for statistical analysis. The overall genotyping call rate achieved for the study cohort was >98.5%. Furthermore, a random 10% subset of samples was subjected to independent repeat genotyping, resulting in 100% duplicate concordance (zero experimental error rate).

Genotype quality was further assessed by testing Hardy–Weinberg equilibrium (HWE) in the control group using the Pearson chi-square goodness-of-fit test, and no significant deviation from HWE was observed, providing additional support for the reliability of the genotyping data.

### 2.7. Statistical Analysis

Statistical analyses were performed using IBM SPSS Statistics version 21.0 (IBM Corp., Armonk, NY, USA). Sample size estimation was conducted using OpenEpi version 6 (Centers for Disease Control and Prevention, Atlanta, GA, USA), assuming a two-sided confidence level of 95%, a statistical power of 80%, a 1:1 case-to-control ratio, an expected exposure frequency of 50% among controls, and a minimum detectable odds ratio of 1.8.

Prior to statistical comparisons, the distribution of continuous variables was first assessed for normality using the Shapiro–Wilk test to determine the selection of parametric or non-parametric analytical methods. Variables with approximately normal distribution are presented as mean ± standard deviation (SD) and were compared using the independent-samples *t*-test for two-group comparisons or one-way analysis of variance (ANOVA) for comparisons involving more than two genotype groups. Variables with a non-normal distribution are presented as median (interquartile range [IQR]) and were analyzed using the Mann–Whitney U test or the Kruskal–Wallis test, as appropriate. Categorical variables are expressed as frequencies and percentages and were compared using Pearson’s chi-square test or Fisher’s exact test when expected cell counts were <5.

Hardy–Weinberg equilibrium (HWE) was evaluated in the control group using Pearson’s chi-square goodness-of-fit test as a quality-control measure for genotyping accuracy. Genotype and allele frequencies were compared between cases and controls under codominant, dominant, recessive, and allelic genetic models. To account for multiple testing across the inheritance models, Bonferroni correction was applied, and both the nominal and Bonferroni-adjusted *p* values are presented (Supplementary Table S1).

The independent association between the *MIA3* rs17465637 polymorphism and coronary artery disease was evaluated using multivariable logistic regression analysis after adjustment for relevant clinical covariates. Adjusted odds ratios (aORs) and corresponding 95% confidence intervals (CIs) were calculated. All statistical tests were two-sided, and a *p* value < 0.05 was considered statistically significant.

The multivariable model was constructed using clinically relevant covariates selected a priori based on established cardiovascular risk factors to estimate adjusted associations rather than to develop a predictive model.

## 3. Results

### 3.1. Clinical and Biochemical Data

This case–control study research approach where it included Saudi individuals as 200 control subjects and 200 CAD patients. Patients with CAD had an average age of 59.3 (SD of 9.7), and 65.4% of them were men. In the control group, which included 59.5% male subjects, the subjects were age matched. The study participants’ clinical and biochemical characteristics are displayed in (Table 1). Regarding weight, body mass index, blood pressure at both the diastolic and systolic points, blood glucose level, and all of the lipid parameters assessed, there was a highly significant difference between the patients and controls. Recruitment from two tertiary hospitals reduced—but could not eliminate—the possibility of regional population structure within Saudi Arabia.

### 3.2. Comparison of Risk Factors and Comorbidities

According to the study’s assessment of risk variables, 66.5% of CAD cases had dyslipidemia, 70% had hypertension, and 59.5% of cases had diabetes. A statistical analysis revealed that there were notable differences in obesity, dyslipidemia, smoking, diabetes, hypertension, and lack of exercise between the patients and controls (Table 2). A review and presentation of previous cardiovascular events among the cases can be found in Table 2.

### 3.3. Genotype and Allele Frequencies in the Study Population

Genotype frequencies in the control group were consistent with Hardy–Weinberg equilibrium (χ^2^ = 0.164, *p* = 0.685). The genotyping for C/*A* (Mutant/Wild) alleles using StepOne RT-PCR shows three clusters for the three possible genotypes CC, AC and AA. The ORs, which indicate an increased risk of CAD in individuals with the CC genotype (OR = 1.89, 95% CI: 0.85–4.19) and heterozygous genotype (OR = 1.45, 95% CI: 0.64–3.28), with *p*-values of 0.112 and 0.368, respectively, are presented along with the genotypic and allelic frequencies of the studied SNP (Table 3). The risk of developing CAD is higher in subjects with the C allele (OR: 1.35, 95% CI: 0.98–1.85, *p* = 0.064).

Bonferroni adjustment was applied across the five prespecified inheritance-model analyses (Supplementary Table S1). None of the exploratory inheritance-model associations remained statistically significant after correction for multiple comparisons.

### 3.4. Multivariate Logistic Regression Analysis

Our research revealed that the SNP rs17465637, smoking, and inactivity were all independent predictors of CAD when age, gender, and the impact of other risk variables including lipid profile were considered (Table 4).

### 3.5. Genotype Distribution Among Different Risk Factors

The three genotypes in the case group showed significant differences in modifiable risk factors for CAD, including smoking, dyslipidemia, and lack of exercise (*p* = 0.03, 0.02, and 0.002, respectively). Across genotypes, gender—a risk factor that cannot be changed—did not differ significantly. Diabetes and hypertension, two controllable variables, did not change substantially amongst the three genotypes (Table 5).

### 3.6. Lipid Profile Stratification by Genotype

As an exploratory secondary analysis intended to generate hypotheses, lipid profiles were stratified according to MIA3 rs17465637 genotypes within the CAD cohort. The CC genotype exhibited the highest mean concentrations of total cholesterol, triglycerides, and LDL-C, together with the lowest HDL-C levels (all *p* < 0.001). Compared with carriers of the wild-type AA genotype, individuals with the heterozygous AC genotype also demonstrated significantly higher total cholesterol and lower HDL-C concentrations (Table 6). These genotype-stratified findings are descriptive and should be interpreted as exploratory observations rather than evidence of causal genotype effects.

### 3.7. Echocardiographic Characteristics According to Genotype

Exploratory genotype-stratified analyses of echocardiographic parameters were performed within the CAD cohort (Table 7). Carriers of the CC genotype demonstrated significantly greater left ventricular internal end-systolic diameter than individuals with the AC/AA genotypes (38.7 ± 6.7 vs. 37.6 ± 8.1 mm, *p* < 0.05). Similarly, left ventricular internal end-diastolic diameter was greater among CC carriers (55.4 ± 5.7 vs. 53.7 ± 6.1 mm, *p* < 0.05). No significant differences were observed in left ventricular end-systolic volume, left ventricular end-diastolic volume, or left ventricular ejection fraction. Given that these analyses were conducted only in patients with CAD, the observed associations should be considered hypothesis-generating and should not be interpreted as establishing causal relationships between the MIA3 genotype and cardiac remodeling.

## 4. Discussion

Genome-wide association studies have, over the last two decades, reshaped how coronary artery disease (CAD) is understood, moving the field away from a purely lipid-centred model toward one in which hundreds of common variants of small individual effect act cumulatively, often through vascular and inflammatory pathways that have little direct connection to cholesterol handling [22]. The *MIA3* locus on chromosome 1q41, first identified in a genome-wide scan of early-onset myocardial infarction and subsequently confirmed in independent European cohorts, is among the earliest and most durable examples of this shift [23,24,25]. Despite this, most of what is known about *MIA3* rs17465637 comes from European, East Asian, and South Asian samples; data from the Arabian Peninsula have been essentially absent. The present case–control study addresses that gap directly, examining rs17465637 in a Saudi population and asking not only whether the variant associates with CAD but also whether it correlates with lipid and echocardiographic phenotypes that might help explain how it contributes to disease.

Overall, our results point in the same direction as prior work without reaching the same level of statistical certainty. The CC genotype and the C allele were both more frequent among cases than controls, yet none of the inheritance models tested survived Bonferroni correction. Taken at face value, this could be read as a failure to replicate. A more accurate reading, in our view, is that the study was underpowered to detect an effect of the size typically reported for this variant, which in most large studies has hovered around an odds ratio of 1.10–1.20 [26]—a magnitude that candidate-gene studies enrolling a few hundred participants per arm are rarely equipped to resolve with confidence [27]. Although unadjusted genotype comparisons did not remain significant after multiple-testing correction, the association persisted after multivariable adjustment, suggesting that the observed relationship may be independent of conventional cardiovascular risk factors [22].

In patients with established coronary artery disease (CAD), lower diastolic blood pressure (DBP) is frequently observed and typically reflects impaired vascular function or advanced disease severity. Accordingly, DBP was included in the multivariable model exclusively as a baseline hemodynamic covariate to control for potential confounding, rather than to imply an independent biological mechanism. Crucially, the observed association must not be interpreted as evidence of a causal protective or harmful effect of DBP itself. Rather, its inclusion serves strictly as a conservative adjustment to isolate and preserve the independent association between the *MIA3* rs17465637 variant and CAD risk [28,29,30].

This pattern mirrors much of the existing literature. The variant was first linked to CAD and myocardial infarction in early-onset European cohorts [23], with the C allele subsequently identified as the risk allele in the German myocardial infarction family study, where haplotype analysis showed that two haplotypes tagging the risk region carried increased odds of infarction [24]. An independent Caucasian cohort from Cleveland reproduced the association after adjustment for standard risk factors [25], and a meta-analysis combining more than fifteen thousand participants of Chinese Han origin confirmed a significant, if modest, effect on both CAD and myocardial infarction, with an odds ratio close to 1.11 [26]. South Asian studies have generally supported the same direction of effect, whether examining rs17465637 in isolation or as part of a combined genetic risk score alongside other GWAS-derived CAD variants [31,32]; in Pakistani cohorts, the risk allele frequency was significantly higher among patients with premature CAD and correlated with an unfavorable balance between pro- and anti-inflammatory cytokines [33]. Not every replication attempt has succeeded. A Spanish study in patients with rheumatoid arthritis found only a non-significant trend toward association with subclinical atherosclerosis [34], and an Iranian case–control study found no significant relationship between rs17465637 and CAD risk, even though the same dataset identified significant associations for other candidate variants [35]. A separate analysis using epistasis-detection methods found that rs17465637 interacts statistically with a variant near TMEM106B, raising the possibility that its contribution to CAD risk depends partly on genetic background rather than acting alone [36]. Set against this uneven but broadly consistent body of work, our findings sit comfortably within the expected range: a real but small effect, visible after adjustment, easy to miss in a genotype-only comparison at moderate sample size.

Several factors likely explain why primary genotype analysis fell short of significance while the international literature, taken as a whole, supports an association. Sample size is the most obvious limitation: with an effect size in the 1.10–1.20 range, adequate statistical power typically requires several thousand participants per group, well beyond what a single-centre study can enrol. Population-specific factors probably contribute as well. Allele frequencies and linkage disequilibrium structure around 1q41 differ across ancestries, and the Saudi population, despite regional homogeneity, carries a demographic history shaped by relatively high rates of consanguinity and localized founder effects that can influence both allele frequencies and the reliability of tagging SNPs [37]. Because genome-wide ancestry-informative markers and principal component analysis were not available in the present study, residual population stratification cannot be fully excluded, even though recruitment was restricted to self-reported Saudi nationals from a single geographic region using consistent eligibility criteria. Finally, the substantial burden of cardiometabolic risk factors documented across Gulf populations may compress the genetic signal by pushing both cases and controls toward a shared, environmentally driven phenotype [38].

### 4.1. Biological Interpretation and Clinical Relevance

Mechanistically, rs17465637 sits within an intron of MIA3, the gene encoding TANGO1, a transmembrane protein positioned at endoplasmic reticulum exit sites. TANGO1 does not act alone: it recruits its paralogue cTAGE5 to form a docking complex that links inner-layer COPII coat components to bulky cargo, most notably procollagen, and without this complex the export of large collagen molecules from the endoplasmic reticulum is markedly impaired [39]. TANGO1 also organizes docking sites between the endoplasmic reticulum and Golgi that support the secretion of other cargo types, a role first demonstrated in the formation of secretory granules in the Drosophila salivary gland [40], and more recent work in cultured human cells shows that its loss disrupts far more than collagen trafficking, destabilizing the entire ER–Golgi interface and reducing secretion of many protein classes, not only large cargo [41]. The physiological stakes of this pathway are illustrated starkly by loss-of-function models: mice and a reported human case lacking functional TANGO1 show near-complete failure of skeletal mineralization, underscoring how central regulated collagen export is to connective tissue formation [42], and comparable phenotypes have been described in zebrafish and medaka fish, where disruption of individual TANGO1 isoforms produces skeletal and cartilage abnormalities even when partial functional redundancy allows survival [43,44]. A related pathogenic variant has even been described outside conventional laboratory models, in a dog breed with an inherited collagen-trafficking disorder affecting teeth, bone, and retina, illustrating how conserved this export machinery is across species [45]. None of this speaks directly to rs17465637 itself, which is intronic and does not alter the TANGO1 amino acid sequence, but it does establish a plausible route by which regulatory changes at this locus—through altered splicing, expression level, or linkage with an as-yet-unidentified functional variant—could influence vascular collagen handling and, downstream, the architecture of the atherosclerotic plaque.

That biological plausibility gains some support from the phenotypic associations observed here. Carriers of the CC genotype had a less favorable lipid profile, with higher total and LDL cholesterol and lower HDL cholesterol than AC or AA carriers. This should be interpreted cautiously: a case–control study cannot establish whether MIA3 variation directly perturbs lipid metabolism or whether the association reflects some shared upstream influence, and no prior study of this SNP has reported a comparably direct lipid effect. Even so, the direction is consistent with a locus that, whatever its precise mechanism, tracks with a more atherogenic overall profile. The echocardiographic findings—larger left ventricular internal dimensions in both systole and diastole among CC carriers—extend this picture beyond susceptibility into the remodeling that follows disease onset. Left ventricular geometry after ischemic injury is shaped substantially by scar formation and extracellular matrix turnover, processes in which fibroblast-derived collagen deposition plays a tightly regulated central role; recent work on infarct healing shows that manipulating collagen and matrix-remodeling pathways in cardiac fibroblasts can either worsen or protect against adverse remodeling [46], and structurally related matrix-regulatory proteins have been shown to influence atherosclerotic plaque stability by modulating collagen content directly [47,48]. Against this backdrop, a variant plausibly connected to collagen export is a reasonable, if still speculative, candidate contributor to both vascular and myocardial structural change.

### 4.2. Clinical Implications and Future Perspectives

The failure of the primary genotype associations to survive Bonferroni correction deserves comment in its own right, because it reflects a pattern seen throughout the candidate-gene literature, not just for MIA3. The broader lesson from the transition between the candidate-gene era and genome-wide association studies is that most individually significant candidate-gene findings failed to replicate once adequately powered, appropriately corrected genome-wide studies became available, largely because early studies underestimated how small the true effect sizes of common variants are and how easily modest samples generate false positives—or, just as often, miss real but small effects once multiple-testing correction is applied [46]. Contemporary large-scale CAD genetics bears this out: multi-ancestry polygenic risk scores built from more than 269,000 cases and 1.1 million controls show that no single variant, including well-established loci such as MIA3, contributes more than a small fraction of overall genetic risk; predictive power comes from aggregating hundreds of thousands of variants rather than from any one locus in isolation [49], and large biobank-scale meta-analyses continue to add further loci at this same modest scale of effect [50]. Read in this light, the failure to reach genome-wide-style significance after correction is not a refutation of the international evidence but an expected consequence of studying a single variant, in a single population, at moderate sample size.

From a translational standpoint, these findings do not support using MIA3 rs17465637 in isolation as a clinical risk marker, and they were never likely to. Individual GWAS-derived CAD variants, almost without exception, have limited standalone predictive value; their utility emerges once they are combined into polygenic risk scores and layered onto conventional clinical risk assessment [22,49]. What this study adds is population-specific groundwork: confirmation that the direction of effect at this locus is consistent with international reports, together with early evidence that its downstream phenotypic correlates—lipid profile and ventricular geometry—are detectable even in a moderate Saudi sample. Both are prerequisites before this or any other variant could reasonably be incorporated into a genetic risk score intended for a Middle Eastern population, given that polygenic scores built and validated in European cohorts are known to lose predictive accuracy when applied to genetically distinct populations [49]. Comparable candidate-gene work from other Saudi and regional groups, spanning genes as varied as those at 9p21, vitamin D metabolism, and inflammatory signaling, points to the same conclusion: population-specific replication is a prerequisite, not an afterthought, before genetic findings established elsewhere can be assumed to generalize to Gulf populations [51,52,53,54,55].

### 4.3. Study Strengths

This study has several strengths. To our knowledge, it is the first to examine MIA3 rs17465637 in a Saudi cohort, adding a population that has been largely absent from the international CAD genetics literature. CAD status was confirmed angiographically rather than by self-report, reducing the risk of phenotypic misclassification that affects genetic epidemiology. The analysis extended beyond a simple genotype comparison by incorporating biochemical and echocardiographic phenotyping, allowing exploration of downstream correlates of the variants rather than testing disease association alone, and the multivariable regression framework made it possible to isolate the genetic signal from major established risk factors rather than reporting an unadjusted association.

### 4.4. Study Limitations

This targeted candidate-gene approach serves as a necessary, population-specific baseline validation. Rather than replacing polygenic risk scores, validating the independent effect of rs17465637 is a crucial prerequisite before this locus can be safely integrated into localized Middle Eastern cardiovascular risk calculators.

The limitations are equally real and should temper how the findings are used. The sample, while reasonable for a single-centre candidate-gene study, remains modest for detecting an effect size in the 1.10–1.20 range, and the AA genotype group in particular was too small to support confident subgroup analysis. Recruitment from two referral hospitals in western Saudi Arabia, without genome-wide ancestry data or principal component analysis, leaves residual population stratification as a possibility that cannot be excluded. The study examined a single SNP in isolation rather than haplotypes, structural variants, or the broader polygenic background, and no functional experiments were performed, so the mechanistic link between rs17465637 and collagen trafficking or vascular remodeling remains inferential, built from separate strands of evidence rather than direct measurement in this cohort.

Formal logistic regression diagnostic procedures, including goodness-of-fit and multicollinearity assessments, were not performed and therefore could not be reported; however, the regression model was specified using predefined clinically relevant covariates to estimate adjusted associations.

Finally, the case–control design cannot establish causality or temporality, and residual confounding from unmeasured dietary, environmental, or epigenetic factors cannot be ruled out.

## 5. Conclusions

Taken together, these findings support MIA3 rs17465637 as a biologically plausible, population-relevant candidate within the polygenic architecture of coronary artery disease among Saudi individuals, rather than as a validated standalone marker. The primary genotype and inheritance-model analyses showed only a trend toward increased CC genotype and C allele frequency among patients that did not survive correction for multiple comparisons, yet the variant remained independently associated with CAD after adjustment for conventional risk factors and correlated with a less favourable lipid profile and greater left ventricular dimensions among CC carriers. Replication in larger, multicentre Saudi and Gulf cohorts, ideally incorporating genome-wide genotyping to address population stratification, together with functional work examining how this intronic variant affects MIA3 expression and collagen export, would help clarify whether the associations observed here reflect a direct contribution to disease or a marker in linkage with an as-yet-unidentified causal variant nearby. Integration of MIA3 with other validated cardiovascular susceptibility loci into polygenic risk models may ultimately support more individualized cardiovascular risk assessment for this and other underrepresented Middle Eastern populations.

## Figures and Tables

**Table 1:** Baseline Physiological and Biochemical Characteristics of Cases and Controls.

Variable	Cases *n* = 200 *n* (%)	Controls *n* = 200 *n* (%)	*p*
Age (y) *	59.3 ± 9.7	58.7 ± 9.3	0.16
Gender (male *n*, %) #	206 ± 65.4%	119 ± 58%	0.055
(a) The clinical examination			
BMI kg/m^2^ *	30.1 ± 4.11	27.3 ± 6.49	**0.03**
Body weight, kg *	82.6 ± 9.1	75 ± 6.8	**0.03**
Waist circumference, cm *	100 ± 7.8	84.3 ± 5.1	**0.04**
Systolic BP mmHg *	138.47 ± 4.7	122.03 ± 2.8	**0.00**
Diastolic BP mmHg *	89.84 ± 2.6	74.25 ± 3.5	**0.01**
Resting Pulse/min *	78 ± 10	77 ± 8	0.06
(b) The laboratory investigation			
Fasting Glucose mmol/L **	7.840 ± 0.217	5.076 ± 0.228	**0.02**
Total Cholesterol mmol/L *	5.725 ± 1.051	4.601 ± 1.037	**0.00**
HDL mmol/L *	0.934 ± 0.290	1.000 ± 0.268	**0.01**
Total/HDL cholesterol ratio *	6.1 ± 1.9	4.5 ± 1.7	**0.00**
LDL mmol/L **	3.065 ± 1.316	2.377 ± 0.835	**0.00**
Triglycerides mmol/L *	2.531 ± 0.740	2.161 ± 0.580	**0.00**

BP, Blood pressure; * mean (SD)/Student *t*-test; ** median (IQR)/Mann–Whitney U test. # frequency(percentage)/χ^2^ test; “*p*” in bold means a statistically significant difference.

**Table 2:** Baseline Risk Factors and Comorbidities of Cases Group.

Risk Factor, *n* (%)	Cases *n* = 200 *n* (%)	Controls *n* = 200 *n* (%)	*p* *
Sedentary life	133 (66.5)	66(33.0)	**0.02**
Overweight	147 (73.3)	124 (60.5)	**0.04**
Dyslipidemia	132 (66.5)	98 (47.8)	**0.03**
Smoking, current + ever	111 (55.5)	72 (35.1)	**0.01**
Hypertension	140 (70.0)	86(44.3)	**0.03**
Diabetes	119 (59.5)	85 (42.5)	**0.01**
**Comorbidities ** ***n*** ** (%)**			
Previous myocardial infarction	33 (16.5)	------	
Previous CABG	15 (7.5)	------	
Previous PCI	22 (11.0)	------	
Previous stroke	11 (5.1)	------	
Peripheral vascular disease	19 (9.5)	------	

CABG: coronary artery bypass graft; PCI: percutaneous coronary intervention * χ^2^ test; “*p*” in bold means a statistically significant difference.

**Table 3:** Comparison of rs17465637 Genotype and Allele Frequencies between CAD/PMI Cases and Controls.

Variable	Coronary Disease *n* = 100, *n* (%)	Post-Myocardial Infarction *n* = 100, *n* (%)	Controls *n* = 200, *n* (%)	OR (95% CI)	*p*-Value
Genotypes					
AA	8 (8.0)	3 (3.0)	18 (9.0)	Reference	
AC	36 (36.0)	35 (35.0)	80 (40.0)	1.45 (0.64–3.28)	0.368
CC	56 (56.0)	62 (62.0)	102 (51.0)	1.89 (0.85–4.19)	0.112
Alleles					
A	52 (26.0)	41 (20.5)	116 (29.0)	Reference	
C	148 (74.0)	159 (79.5)	284 (71.0)	1.35 (0.98–1.85)	0.064

Notes: Hardy–Weinberg equilibrium in controls: χ^2^ = 0.164, *p* = 0.685; genotype distribution was consistent with HWE. Primary genetic association calculations were performed using the combined case–cohort (CAD + PMI; *n* = 200) versus controls (*n* = 200). CAD and PMI subgroup counts are presented descriptively to preserve the submitted table structure.

**Table 4:** Corrected Multivariable Logistic Regression Analysis of Factors Associated with CAD.

Variable	β	SE	Adjusted OR	95% CI Lower	95% CI Upper	*p*-Value
rs17465637 (C-allele dosage)	0.832	0.305	2.298	1.263	4.182	0.006
BMI (kg/m^2^)	0.012	0.022	1.012	0.968	1.057	0.599
Smoking	0.65	0.259	1.916	1.153	3.184	0.012
Physical inactivity	−0.55	0.406	0.577	0.26	1.279	0.175
Systolic BP	0.159	0.04	1.173	1.084	1.268	<0.001
Diastolic BP	−0.182	0.067	0.834	0.731	0.95	0.006
Blood glucose	0.024	0.005	1.024	1.014	1.034	<0.001
Triglycerides	−0.006	0.003	0.994	0.989	1	0.04
Total cholesterol	0.024	0.008	1.024	1.008	1.04	0.003
LDL-C	−0.016	0.009	0.984	0.966	1.002	0.089
HDL-C	−0.002	0.014	0.998	0.971	1.026	0.888

Notes: Values were recalculated from the manuscript dataset. The model included rs17465637 C-allele dosage, BMI, smoking, physical inactivity, systolic BP, diastolic BP, blood glucose, triglycerides, total cholesterol, LDL-C, and HDL-C. Complete-case *n* = 360. Adjusted OR = exp(β).

**Table 5:** Risk Factors Distribution Across Genotypes.

	CC *n* = 118	AC *n* = 71	AA *n* = 11	*p* *
Age	65.4 (10.9)	59.4 (10.7)	59.93 (10.3)	0.60
BMI (kg/m^2^)	29 (3.4)	29.2 (4.3)	29.7 (3.7)	0.51
Gender (male *n*, %)	75 (63.6)	24 (33.8)	8 (72.7)	0.65
Systolic BP mmHg	147.5 (10.4)	145.4 (8.03)	143.1 (10.4)	0.37
Diastolic BP mmHg	93.3 (8.5)	91.3 (7.4)	89.3 (5.8)	0.28
Pulse/min	80.8 (2.3)	79.3 (3.6)	78.4 (2.8)	0.16
Blood Glucose mg/dL	137.1 (2.9)	138.1(2.4)	139.7 (1.9)	0.44
Sedentary life, *n* (%)	39 (33.1)	6 (8.45)	2 (18.8)	**0.002**
Overweight, *n* (%)	71 (60.2)	29 (40.8)	8 (72.7)	0.56
Dyslipidemia, *n* (%)	93 (78.8)	27 (38.0)	5 (45.5)	**0.02**
Smoking, *n* (%)	88 (74.5)	19 (26.8)	5 (45.5)	**0.03**
Hypertension, *n* (%)	88 (74.5)	25 (35.2)	9 (81.8)	0.66
Diabetes, *n* (%)	79 (66.9)	21 (29.6)	8 (72.7)	0.28

* χ^2^ and ANOVA tests “*p*” in bold means a statistically significant difference.

**Table 6:** Lipid Profile Characteristics Distribution Across Genotypes.

	TC (mg/dL)	LDLc (mg/dL)	HDLc (mg/dL)	TG (mg/dL)
Genotypes				
CC	6.758 ± 1.089 **	3.600 ± 0.528 **	0.596 ± 0.082 **	3.025 ± 0.478 **
AC	6.321 ± 0.807 *	2.910 ± 0.445	0.841 ± 0.119 *	2.387 ± 0.137
AA	5.369 ± 0.320	2.253 ± 0.313	1.237 ± 0.160	2.319 ± 0.177

* ANOVA test *p* value ˂ 0.05. ** *p* value ˂ 0.001.

**Table 7:** Echocardiographic Characteristics Distribution Across Genotypes.

	LVEF %	LVIDs (mm)	LVIDd (mm)	LVESV (mL)	LVEDV (mL)
Genotypes					
CC	55.8 ± 10.1	38.7 ± 6.7 *	55.4 ± 5.7 *	48.8 ± 27.1	115 ± 38.1
AC/AA	57.3 ± 11.3	37.6 ± 8.1	53.7 ± 6.1	47.2 ± 25.2	110.2 ± 36.7

* ANOVA test *p* value ˂ 0.05. LVEF—Left ventricular ejection fraction LVIDs and LVIDd—Left ventricular internal diameter end systole and end diastole LVESV and LVEDV—Left ventricular end systolic and diastolic volume.

## Data Availability

Upon reasonable request, the corresponding author can supply the datasets utilized and/or analyzed in the current work due to ethical issues.

## Supplementary Materials

The following supporting information can be downloaded at https://www.mdpi.com/article/10.3390/diagnostics16162489/s1, Table S1: Association of rs17465637 with CAD under Different Genetic Inheritance Models.

## List of Abbreviations

AA	homozygous Adenine genotype
AC	heterozygous genotype
BMI	body mass index
CC	homozygous Cytosine genotype
CI	Confidence interval
CAD	Coronary artery disease
DNA	Deoxycarboxy nucleic acid
ECG	Electrocardiogram
EDTA	Ethylenediaminetetraacetic acid
GWAS	Genome-wide association studies
IFCC	International Federation of Clinical Chemistry and Laboratory Medicine
KAMC	King Abdullah Medical City
LV	Left ventricular
MIA3	Melanoma-inhibitory family member gene
OR	Odd’s ratio
PMI	Post-myocardial infarction
RT-PCR	Real-time polymerase chain reaction
SNPs	Single-nucleotide polymorphisms
WHO	World Health Organization
