# Supplementary material for: Evaluating the Role of MIA3 Variant rs17465637 in Coronary Artery Disease: A Comprehensive Case–Control Analysis
Source: Diagnostics (Basel). 2026 Aug 7;16(16):2489. doi: 10.3390/diagnostics16162489 (PMC13511093; doi:10.3390/diagnostics16162489)
Supplement: Supplementary file 1 [file diagnostics-16-02489-s001.zip › diagnostics-4401061-supplementary.pdf]

Supplementary Table S1. Association of rs17465637 with CAD under Different Genetic Inheritance Models

Genetic model	Comparison	Cases exposed	Controls exposed	Reference group	OR	95% CI lower	95% CI upper	P-value	Bonferroni-adjusted P	Interpretation
Codominant	AC vs AA	71	80	Cases ref=11; Controls ref=18	1.45	0.64	3.28	0.368	1	Not significant after Bonferroni correction
Codominant	CC vs AA	118	102	Cases ref=11; Controls ref=18	1.89	0.85	4.19	0.112	0.558	Not significant after Bonferroni correction
Dominant	AC+CC vs AA	189	182	Cases ref=11; Controls ref=18	1.7	0.78	3.7	0.177	0.886	Not significant after Bonferroni correction
Recessive	CC vs AA+AC	118	102	Cases ref=82; Controls ref=98	1.38	0.93	2.05	0.108	0.539	Not significant after Bonferroni correction
Additive / allelic	Per C allele	307	284	Cases ref=93; Controls ref=116	1.35	0.98	1.85	0.064	0.321	Not significant after Bonferroni correction

Notes: Bonferroni adjustment was applied across the five tested genetic model comparisons. None of the inheritance models remained significant after Bonferroni correction.
